# Takotsubo Syndrome Occurring after mRNA COVID-19 Vaccination in a Patient with Graves’ Disease

**DOI:** 10.3390/medicina59010094

**Published:** 2022-12-31

**Authors:** Yen-Chi Chen, Gen-Min Lin, Eiki Takimoto, Chin-Sheng Lin, Wen-Yu Lin, Chun-Hsien Wu, Chia-Luen Huang, Cheng-Chung Cheng, Shu-Meng Cheng, Shih-Hua Lin, Issei Komuro, Pang-Yen Liu

**Affiliations:** 1 Division of Gastroenterology and Hepatology, Department of Medicine, Tri-Service General Hospital, National Defense Medical Center, Taipei City 11490, Taiwan; 2 Department of Medicine, Hualien Armed Forces General Hospital, Hualien City 97144, Taiwan; 3 Department of Cardiovascular Medicine, Graduate School of Medicine, Faculty of Medicine, The University of Tokyo, Tokyo 113-0033, Japan; 4 Division of Cardiology, Johns Hopkins Medical Institutions, Baltimore, MD 21287, USA; 5 Division of Cardiology, Department of Medicine, Tri-Service General Hospital, National Defense Medical Center, Taipei City 11490, Taiwan; 6 Division of Endocrinology and Metabolism, Department of Medicine, Tri-Service General Hospital, National Defense Medical Center, Taipei City 11490, Taiwan; 7 Division of Nephrology, Department of Medicine, Tri-Service General Hospital, National Defense Medical Center, Taipei City 11490, Taiwan

**Keywords:** COVID-19, Moderna COVID-19 vaccine, adverse effects, female, Takotsubo syndrome, Graves’ disease

## Abstract

Cardiovascular events such as myocarditis following mRNA COVID-19 vaccination are increasing. We present a 67-year-old postmenopausal woman with Takotsubo Syndrome and Graves’ disease after mRNA COVID-19 vaccination. She developed chest pain and shortness of breath one week after vaccination. An electrocardiogram revealed ST elevation in the precordial leads. Coronary angiography revealed the absence of obstructive coronary artery disease, and the left ventriculography showed a typical feature with apical ballooning. Laboratory workup showed the elevation of free T4 and thyrotropin receptor antibodies. It was presumed that Takotsubo Syndrome and Graves’ disease were probably related to the COVID-19 mRNA vaccination. The patient was treated with low-dose bisoprolol, diuretics, carbimazole, and steroid and discharged uneventfully. The mRNA COVID-19 vaccination is still safe and effective to defend against COVID-19 pandemic. However, clinicians should be aware of the possible cardiovascular adverse events other than myocarditis following vaccination.

## 1. Introduction

As of January 2022, the Coronavirus disease (COVID-19) pandemic has affected about 352,700,000 patients worldwide. Vaccination against the disease has also been globally implemented with around nine to ten billion vaccine doses administrated. However, cardiovascular events after vaccine administration have been increasingly reported [1].

Takotsubo syndrome is characterized by transient regional left ventricle systolic dysfunction. It mimics myocardial infarction but with the absence of angiographic evidence of obstructive coronary artery disease or acute plaque rupture. Usually, the regional wall motion abnormality extends beyond the territory perfused by a single epicardial coronary artery. Takotsubo syndrome is much more common in postmenopausal women, but its pathogenesis is not well elucidated. Vaccination can be a possible cause due to an immune inflammatory response. Herein, we present a case of Takotsubo Syndrome and Graves’ disease following mRNA COVID-19 vaccination.

## 2. Case Presentation

A 67-year-old postmenopausal female housekeeper presented to the emergency department with chest pain, palpitation, dyspnea on exertion, nausea, and diarrhea. The patient had no relevant past medical history and was not on any medication. One week earlier, she received her first dose of mRNA-1273 (Moderna) SARS-CoV-2 vaccination.

**Figure 1:**
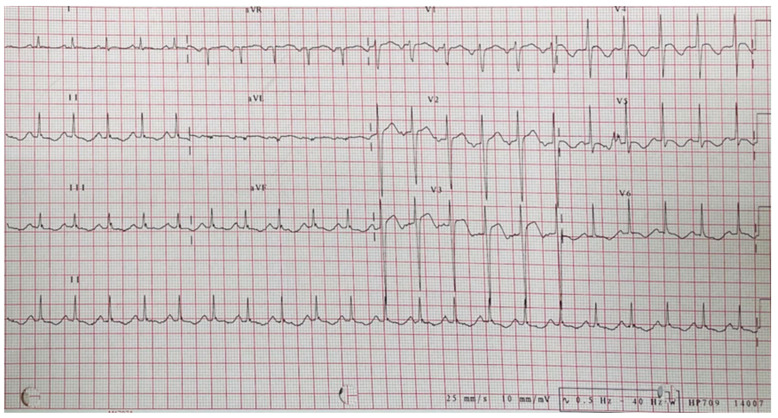
Electrocardiogram showed sinus tachycardia and ST-elevations in leads V2 and V3; ST-depression in leads II, III, aVF, and V4–V6.

On initial evaluation, the patient’s blood pressure was 106/69 mmHg. Her heart rate was 113 beats/min. She was afebrile. Her cardiac auscultation revealed regular and rapid heartbeats with grade 2 systolic murmurs over the left lower sternal border. Her respiratory and abdominal examinations were normal. An electrocardiogram (ECG) revealed sinus tachycardia and ST elevation in the precordial leads (Figure 1). A laboratory workup showed the following: thyroid stimulating hormone 0.005 (normal: 0.27–4.2) μIU/mL, free T4 77.7 (normal 9.3–17) ng/L, thyrotropin receptor antibody 9.19 (normal < 1.75) IU/L, high-sensitivity troponin-I 1298 (normal < 29) mcg/L, and NT-proBNP 3872 (normal: < 125) ng/L. Hyperthyroidism with Graves’ disease was highly suspected.

**Figure 2:**
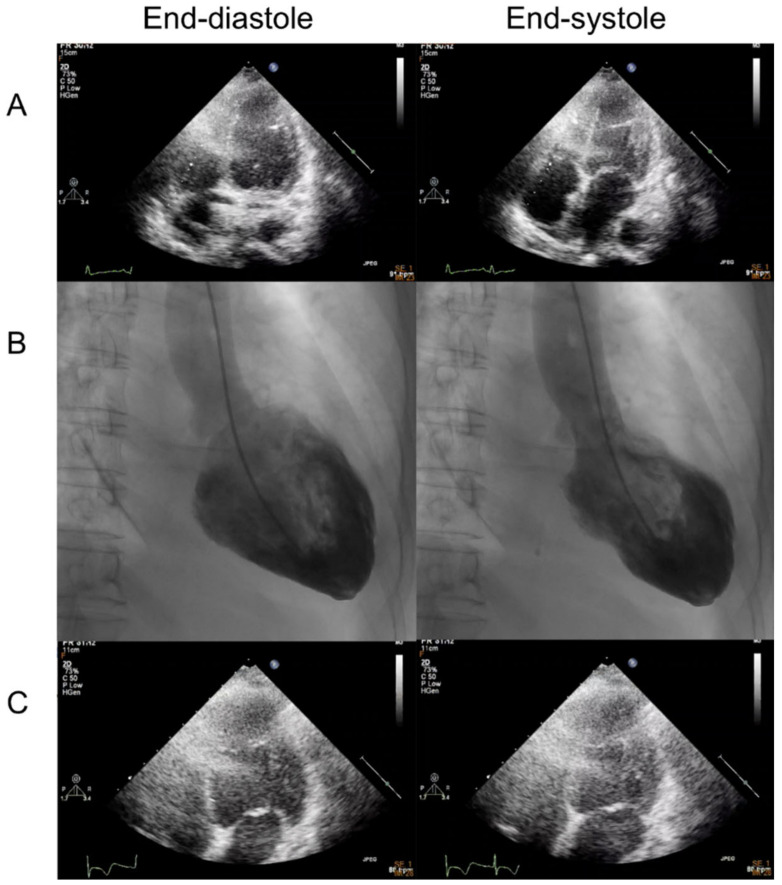
Left ventriculogram and serial echocardiogram of Takotsubo syndrome occurring after COVID-19 mRNA vaccination. (**A**,**B**) Echocardiography (four-chamber images) and left ventriculography at the presentation of Takotsubo syndrome after COVID-19 mRNA vaccination. (**C**) Repeat echocardiography (four-chamber images) revealed an improvement in apical ballooning on day 6. Notice the slow recovery of the negative T wave during the subacute phase (left panel: end-diastolic phase, right panel: end-systolic phase).

**Figure 3:**
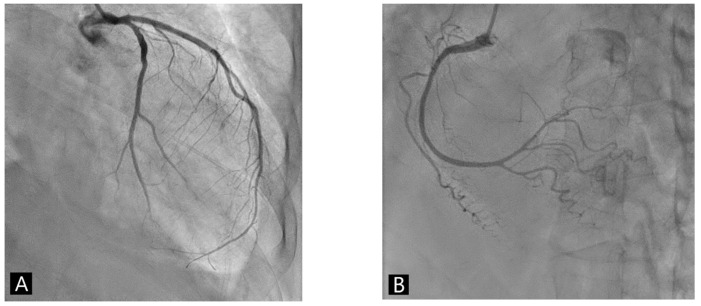
Coronary angiography showed no remarkable atherosclerotic change or stenosis of coronary arteries. (**A**) RAO cranial view. (**B**) LAO cranial view.

Echocardiography showed reduced left ventricular systolic function as an ejection fraction of 45% and akinesia of apex with preserved systolic wall motion in mid-ventricular and basal left ventricle of the heart (Figure 2A and Supplementary Video S1). Coronary angiography revealed the absence of obstructive coronary artery disease (Figure 3). The left ventriculography showed a typical feature with apical ballooning (Figure 2B and Supplementary Video S2).

We administered low-dose bisoprolol and diuretics. She also received carbimazole and steroids. On day 6, a follow-up ECG showed normal sinus rhythm. Repeat transthoracic echocardiography demonstrated the rapid recovery of apical ballooning of the left ventricle without any apical akinesia (Figure 2C and Supplementary Video S3). ECG tracings during echocardiography showed that the negative T wave recovered more slowly during the subacute phase. The transient abnormal left ventricular systolic function, transient ECG changes, and absence of obstructive coronary artery disease were suggestive of Takotsubo syndrome (TTS) and the patient was diagnosed with TTS. The patient was finally discharged with bisoprolol, propylthiouracil (200 mg daily), and prednisolone (5 mg daily).

One week after discharge, the patient was followed up in the outpatient department. There was no chest pain, palpitation, or dyspnea on exertion. The laboratory results showed a thyroid-stimulating hormone level below 0.005 μIU/mL, and free T4 was above 77.7 ng/L. We prescribed bisoprolol and propylthiouracil (200 mg daily) for ongoing hyperthyroidism with Graves’ disease.

## 3. Discussion

The Phase III trial data for the mRNA-1273 SARS-CoV-2 vaccine reported no significant risk of cardiovascular reactions compared to a placebo [2]. However, cases of cardiovascular events after the administration of vaccines including myocarditis, pericarditis, congestive heart failure, and myocardial infarction have been reported increasingly [1].

The present case describes a presentation of typical TTS and Graves’ disease after the first dose of mRNA-1273 SARS-CoV-2 vaccination in a postmenopausal woman. A systemic review has recruited 10 cases with TTS following COVID-19 vaccination between 1 January 2020 and 1 June 2022 [3]. In total, 10 out of 11 patients were female, and 7 of them were older than 60 years old. Chest pain was the most common symptom. The onset of symptoms in all cases was within a week after vaccination. All of the cases were discharged eventually. Among cardiovascular reactions of SARS-CoV-2 vaccination, myocarditis has been documented as a rare complication, especially in young adult and adolescent males [4]. In contrast, SARS-CoV-2 vaccination-associated TTS was exclusively reported in elderly females, such as the present case according to all reports to date, except in the case of a male with comorbid ischemic heart disease reported by Crane et al. (Table 1.) [5,6,7,8,9,10,11,12,13,14].

Although SARS-CoV-2 vaccination-associated myocarditis still could be a possible diagnosis in this case, the clinical features and typical apical ballooning in left ventriculography were strongly suggestive of TTS. The exact mechanisms of TTS after SARS-CoV-2 vaccination remains unclear. It may involve a systemic inflammatory reaction induced by vaccination with the overstimulation of sympathetic signaling. The mRNA-1273 SARS-CoV-2 vaccine encodes the stabilized prefusion SARS-CoV-2 spike protein. SARS-CoV-2 spike protein antibody might have cross-reacted with a variety of tissue antigens along with reacting to SARS-CoV-2 proteins [15]. That could have led to the autoimmune reaction after the administration of vaccines and possible cytokine storm, with symptoms similar to SARS-CoV-2 infection [16]. In addition, six cases of Graves’ disease following SARS-CoV-2 vaccination were reported, and four cases received the BNT162b2 mRNA SARS-CoV-2 vaccine [17].

We believe that SARS-CoV-2 vaccination-associated myocarditis and TTS are within the spectrum of immune response. The prognosis of TTS is distinct from that of myocarditis [18]. Longitudinal studies are needed for evaluating the outcomes of TTS after SARS-CoV-2 vaccination. The postvaccination phenomenon appears after being exposed to adjuvants in vaccines that increase the immune response, and autoimmune/inflammatory syndrome induced by adjuvants [19]. The mRNA SARS-CoV-2 vaccination could directly or indirectly cause TTS in the setting of Graves’ disease in the present case.

Both TTS and Graves’ disease developed after SARS-CoV-2 vaccination in this case, though we could not exclude the causality between TTS and Graves’ disease. A multi-center observational study reported that 4 of 16 cases with TTS revealed subclinical or overt thyrotoxicosis [20]. Thyrotoxicosis can aggravate inotropic and chronotropic responses to catecholamine with the over-regulation of beta-adrenergic receptors in cardiac muscles, which is a possible way to trigger TTS [21]. However, TTS subsided with ongoing Graves’ disease in this case. Nevertheless, we could not rule out the possibility that TTS owing to Graves’ disease was found just coincidentally after SARS-CoV-2 vaccination.

## 4. Conclusions

We report a case in which the concomitant development of Takotsubo Syndrome and Graves’ Disease occurred after mRNA COVID-19 vaccination. The findings of the present report raise the possibility of an association between mRNA SARS-CoV-2 vaccination and TTS, particularly in postmenopausal women. Nevertheless, we still need further studies to establish the pathophysiologic mechanism of SARS-CoV-2 vaccination-associated complications.

## Figures and Tables

**Table 1:** Takotsubo Syndrome Occurring after COVID-19 Vaccination.

Author/Year of Publication	Jani et al. [5]	Boscolo Berto et al. [6]	Toida et al. [7]	Caitlin Stewart et al. 2021 [8]	Fearon et al. [9]	Crane et al. [10]	Vidula et al. [11]	Hiroki Yamaura et al. 2022 [12]	Tedeschi et al. [13]	Ricci et al. [14]	The Present Case
Country	United States	Switzerland	Japan	United Kingdom	United States	Australia	United States	Japan	Italy	Italy	Taiwan
Age, year	65	63	80	50	73	72	60	30	71	54	67
Sex	Female	Female	Female	Female	Female	Male	Female	Female	Female	Female	Female
Comorbidities	Hyperlipidemia	None	Renal sclerosis on maintenance hemodialysis, hypertension	Chronic obstructive pulmonary disease	Chronic kidney disease, hypertension	Ischemic heart disease, hypertension, type 2 diabetes mellitus, ulcerative colitis	Coronary artery disease	None	Congenital Long QT Syndrome	None	Thyrotoxicosis
Vaccine received	mRNA-1273	mRNA-1273	Pfizer-BioNTech	ChadOX1 nCOV-19	mRNA-1273	ChadOX1 nCOV-19	Pfizer-BioNTech	Pfizer-BioNTech	Pfizer-BioNTech	mRNA-1273	mRNA-1273
Doses received	1	1	1	2	NA	1	2	2	1	2	1
Interval after vaccination, days	3	1	1	7	1	4	4	2	<1	<1	7
Symptoms	chest pain, myalgia, nausea, headache	dyspnea and fever	general fatigue and loss of appetite	chest pain	chest pain, shortness of breath, fatigue, and nausea	chest pain, fatigue, myalgias, and fever	chest pain	chest pain and cold sweat	chest pain and shortness of breath	palpitations, asthenia, and intermittent chest tightness	chest pain, palpitation, dyspnea on exertion, nausea, and diarrhea
LVEF (%)	35%	40%	48%	NA	65%	60%	47%	NA	38%	40%	45%
Electrocardiogram (ECG)	Evolving ST-T changes suggestive of inferolateral ischemia	TWI over the inferior/anterior leads	TWI in I, aVL, and V3-6, and a prolonged QTc	Anterior T wave inversion and a prolonged QTc	ST-T changes of inferolateral ischemia and PRWP	First-degree and RBBB without acute or dynamic ischemic changes	Inferolateral TWI	ST-segment depression on the V4-V6 leads	TWI in all leads except for aVL and aVF, and prolonged QTc	PRWP and TWI in inferior leads and from V3 to V6.	ST-T changes of inferior/anterior leads
Troponin (mcg/L)	5.7 (Tn)	3.20 (hsTnT)	2.26 (Tn)	1.66 (Tn)	220 (Tn)	1.86 (Tn)	0.12 (Tn)	1.00 (Tn)	0.34 (hsTnT)	Elevated	1298 (hsTnI)
NT-proBNP (ng/L)	NA	10,180	NA	NA	>70,000	NA	NA	NA	2261	Elevated	3872
Therapy											
beta-blocker	Low-dose metoprolol	NA	None	None	Metoprolol	NA	Metoprolol	None	NA	NA	Low-dose bisoprolol
ACEi/ARB	Lisinopril	NA	None	None	Losartan	NA	Lisinopril	None	NA	NA	None
Length of hospital stay (days)	NA	NA	13	5	8	10	NA	15	NA	NA	6
Outcome	Uneventfully Discharged	Uneventfully Discharged	Uneventfully Discharged	Uneventfully Discharged	Uneventfully Discharged	Uneventfully Discharged	Uneventfully Discharged	Uneventfully Discharged	Uneventfully Discharged	Uneventfully Discharged	Uneventfully Discharged

TWI: T wave inversions, QTc: corrected QT interval, PRWP: poor anterior R wave progression, RBBB: right bundle branch block, Tn: troponin, hsTnI: high-sensitivity troponin-I, hsTnT: high-sensitivity troponin-T, NA: not available, ACEi: angiotensin-converting enzyme inhibitor, ARB: angiotensin-receptor blocker.

## Data Availability

The data presented in this study are available on request from the corresponding author.

## Supplementary Materials

The following supporting information can be downloaded at: https://www.mdpi.com/article/10.3390/medicina59010094/s1, Video S1: echocardiography, Video S2: left ventriculogram, Video S3: repeat echocardiography.
